# Optimal Therapeutic Strategy of Bone Marrow-Originated Autologous Mesenchymal Stromal/Stem Cells for ALS

**DOI:** 10.1093/stcltm/szad095

**Published:** 2024-01-19

**Authors:** Seung Hyun Kim, Ki-Wook Oh, Min-Young Noh, Min-Soo Kwon

**Affiliations:** Department of Neurology, College of Medicine, Hanyang University, Seoul, Republic of Korea; Cell Therapy Center, Hanyang University Hospital, Seoul, Republic of Korea; Department of Neurology, College of Medicine, Hanyang University, Seoul, Republic of Korea; Cell Therapy Center, Hanyang University Hospital, Seoul, Republic of Korea; Department of Neurology, College of Medicine, Hanyang University, Seoul, Republic of Korea; Cell Therapy Center, Hanyang University Hospital, Seoul, Republic of Korea; Department of Pharmacology, Research Institute of Basic Medical Science, School of Medicine, CHA University, CHA Bio Complex, Gyeonggi-do, Republic of Korea

**Keywords:** amyotrophic lateral sclerosis, MSC therapy, ATMPs-GMP, optimal therapeutic strategy

## Abstract

Amyotrophic lateral sclerosis (ALS) is characterized by selective and progressive neurodegenerative changes in motor neural networks. Given the system complexity, including anatomically distributed sites of degeneration from the motor cortex to the spinal cord and chronic pro-inflammatory conditions, a cell-based therapeutic strategy could be an alternative approach to treating ALS. Lessons from previous mesenchymal stromal/stem cell (MSC) trials in ALS realized the importance of 3 aspects in current and future MSC therapy, including the preparation of MSCs, administration routes and methods, and recipient-related factors. This review briefly describes the current status and future prerequisites for an optimal strategy using bone-marrow-originated MSCs to treat ALS. We suggest mandatory factors in the optimized therapeutic strategy focused on advanced therapy medicinal products produced according to Good Manufacturing Practice, an optimal administration method, the selection of proper patients, and the importance of biomarkers.

## Graphical Abstract

**Graphical Abstract:**
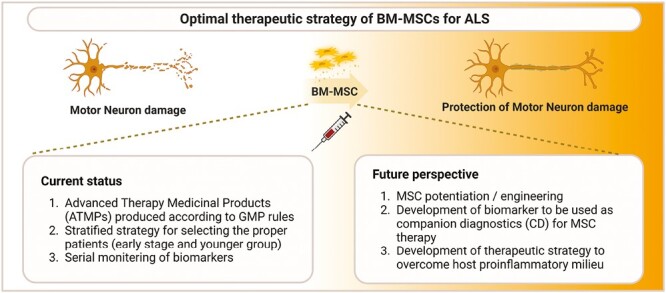

**Significance Statement**

Mesenchymal stromal/stem cell (MSC)-based therapy is a promising strategy to address the complex multifactorial pathomechanisms of motor neuronal death in amyotrophic lateral sclerosis (ALS). For enhanced safety and efficacy, protocols for MSC preparation and manufacturing, administration methods, and recipient selection criteria should be optimized based on the results of well-designed randomized controlled trials and current guidelines. Optimized protocols for the potentiation/engineering of MSCs and the development of reliable biomarkers to predict the pretreatment status of the recipient milieu and the therapeutic response are prerequisites for an innovative therapeutic strategy using MSCs for ALS.

## Introduction

Amyotrophic lateral sclerosis (ALS) is a progressive neurodegenerative disease affecting both the upper and lower motor neuron systems and eventually results in generalized weakness and, ultimately, death due to respiratory failure.^1,2^ With the recent breakthroughs in comprehending the pathogenic mechanisms of ALS, several crucial points have emerged: (1) The identification of novel causative genes has broadened our understanding of ALS, shifting it toward a motor neural network syndrome or multisystem proteinopathy. (2) Recognizing noncell autonomous contributions to the pro-inflammatory central nervous system (CNS) milieu underlying motor neuronal cell death underscores the importance of further investigating immune-inflammatory modulation as a potential clinical therapeutic strategy for neurodegenerative diseases, including ALS. To date, only 3 drugs have been approved by the Food and Drug Administration (FDA): riluzole, edaravone, and AMX0035 (Relyvrio).^3-5^ However, they have modest therapeutic effects.^6-8^

Despite significant progress in linking clinical heterogeneity with the genetic and pathomechanisms of ALS, most previous clinical trials have not yielded successful outcomes.^9,10^ Failed trials, primarily based on single molecular targets, underscore the significance of incorporating multiple molecular targets within a broader therapeutic strategy. In this context, clinical trials using mesenchymal stromal/stem cells (MSCs) have been conducted for 2 decades for ALS.

This review briefly describes the current status and future prerequisites for an optimal MSC therapeutic strategy for ALS. We confined this review to autologous bone marrow-originated MSC (BM-MSC) treatment for ALS and described the current status and prerequisites for an optimal therapeutic strategy. We suggest mandatory factors for the strategy focused on advanced therapy medicinal products (ATMPs) produced according to Good Manufacturing Practice (GMP), optimal administration routes and protocols, biomarker-based stratification of patients, the monitoring of post-treatment effectiveness, and an optimized therapeutic schedule. The future development of such a strategy for ALS, including MSC potentiation with priming methods and genetic engineering and the development of reliable biomarkers to predict the recipient’s milieu, could give hope to patients with ALS.

## Therapeutic Targets for BM-MSCs Based on the Pathogenic Mechanisms of ALS

Despite the diversity of genetic risk factors associated with ALS, neuroinflammatory responses are known to play a pivotal role in the pathophysiology of the disease and are intimately connected with its progression.^11^ Neuroinflammation observed in ALS is fueled by an intricate interplay between the peripheral immune system and the brain’s immune system.^12^ ALS is considered a noncell autonomous disease, where the start and progression of motor neuron degeneration seem to be influenced by interactions among different kinds of cells and the development of a sustained inflammatory environment.^13^

Notably, decreased numbers and activity of regulatory T cells (Tregs) and decreased forkhead box P3 (FoxP3) expression levels in peripheral lymphocytes have been suggested as biomarkers predicting rapid progression and attenuated survival in patients with ALS.^14^ Treg cells have been demonstrated to possess the capacity to induce the functional shifting of microglia toward the alternative, protective phenotype^15-17^ and suppress Type 1 T helper (Th1) lymphocyte proliferation. Microglia, which exist as innate immune cells in the CNS parenchyma as phagocytes, secret various toxic cytokines, as well as tissue-repairing molecules, in a context-dependent manner.^18^

**Figure 1.**
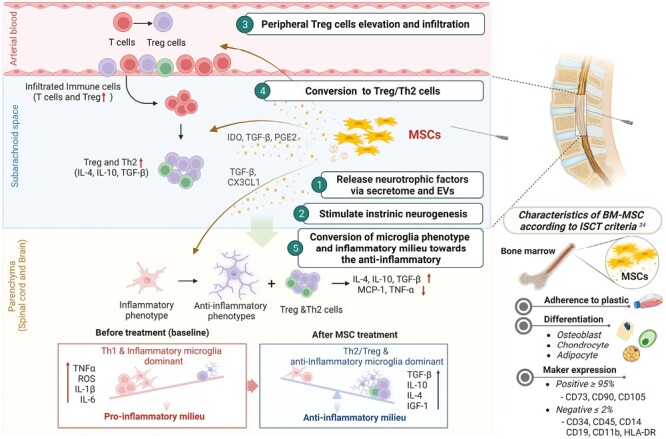
BM-MSCs intrathecally injected for ALS and verified according to the minimum ISCT criteria,^24^ operate through various mechanisms. They release neurotrophic factors, fostering neural health. In cerebrospinal fluid, MSCs increase populations of regulatory T cells and Th2 cells, which move into the CNS, leading to an anti-inflammatory environment marked by anti-inflammatory cytokines, such as interleukin (IL)-4 and IL-10. Additionally, BM-MSCs release balanced transforming growth factor-β (TGF-β) for CNS homeostasis, prompting a transition in microglia from an inflammatory to an anti-inflammatory phenotype. This transformation contributes to an anti-inflammatory environment with balanced IL-4, IL-10, and TGF-β concentrations, promoting CNS homeostasis. BM-MSCs also exhibit diverse immunomodulatory properties and can regulate the onset and progression of ALS by enhancing regulatory T cells and encouraging the development of anti-inflammatory microglia.^25-31^The image was created with BioRender.com.

Given the system complexity and anatomically distributed sites of degeneration from the motor cortex to the spinal cord, cell therapy focused on replacing injured cells and differentiation into motor neurons is challenging.^19^ Furthermore, even if these new motor neurons are integrated into a diseased neural network, they may remain susceptible to the same pathological processes that destroy original motor neurons.^20-23^ Therefore, a therapeutic strategy using MSCs should be focused away from neuronal replacement or reconstruction and toward creating an anti-inflammatory microenvironment. As summarized in Fig. 1, besides direct effects, such as the release of neurotrophic factors and the stimulation of intrinsic neurogenesis, intrathecally administered MSCs have diverse immune inflammatory modulatory efficacy that can regulate the onset and progression of ALS by potentiating regulatory T cells and anti-inflammatory microglia in the CNS environment.^25,26^

## Mandatory Factors in an Optimized Therapeutic Strategy Using MSCs for ALS

Guidelines for clinical trials in ALS emphasize a trial duration of 6-12 months to determine treatment effectiveness and recommend using composite endpoints (survival and function) to increase power.^32^ Recent guidelines also emphasize the importance of excluding genetic and clinical heterogeneity when enrolling subjects and using post hoc analysis of biological markers to identify subgroups of patients who appear to respond better to a specific treatment.^33^ The guidelines also mention the small number of people with ALS, a rare disease, as a barrier to drug development and the need for adaptive platform trials, in which multiple drugs and a common control group can be shared, to address the disadvantage of patients in the control group who do not receive treatment with an investigational drug that is expected to have a therapeutic effect during the trial. Thus, it is crucial to determine an optimal therapeutic strategy within the context of the current guidelines.

Recently published reviews^34,35^ and Cochrane data^36^ regarding stem cell therapy for ALS showed that numerous preclinical and early-stage clinical trials have been conducted. The major clinical trials using MSCs for ALS are summarized in Table 1. The route of administration in human trials has mostly been intrathecal, with the remainder being intrathecal with intramuscular, intravascular, or intraspinal. However, only 2 clinical trials were low risk of bias randomized controlled trial (RCT) designs and met current guidelines (NCT01363401, NCT03280056). A lenzumestrocel phase II RCT (NCT01363401) in which patients received 2 intrathecal injections of autologous BM-MSCs with riluzole or riluzole alone (controls) demonstrated transient positive clinical effects on Amyotrophic Lateral Sclerosis Functional Rating Scale‑Revised (ALSFRS-R) scores with good safety. The NurOwn phase III RCT (NCT03280056) in which patients received 3 intrathecal injections of autologous preconditioned BM-MSCs or placebo did not meet the primary outcome. Subgroup analysis in the early and moderate stages of ALS showed a possible clinical benefit according to ALSFRS-R scores.

**Table 1.** Summary of major MSC clinical trials for ALS.

Trial number	Location/Sponsor	Study design	Treatment arm	Study drug	Delivery route	Result	Reference
NCT01363401	Korea/Corestem	Phase I	Two injections with a 26-day interval (*n* = 7)	Autologous BM-MSCs (Lenzumestrocel, Neuronata-R® Inj)	Intrathecal	Safe	Oh et al.^28^
NCT01051882	Israel/Brainstorm	Phase I/II, open-label	(i) Early-stage ALS group (n = 6) received a single intramuscular injection (ii) Advanced disease group (*n* = 6) received a single intrathecal injection	Autologous preconditioned BM-MSCs (NurOwn®)	Intramuscular or intrathecal	Safe	Petrou et al.^37^
NCT01777646	Israel/Brainstorm	Phase IIa, open-label, dose-escalating	Single injection (*n* = 14)	Autologous preconditioned BM-MSCs (NurOwn®)	Intramuscular and intrathecal	Safe; possible clinical benefit	Petrou et al.^37^
NCT01363401	Korea/Corestem	Phase II, RCT without a sham procedure	Randomized (1:1) (i) Two injections with a 26-day interval (*n* = 32) (ii) Control group (*n* = 27)	Autologous BM-MSCs (Lenzumestrocel, Neuronata-R® Inj)	Intrathecal	Safe; positive clinical effect, reduced pro-inflammatory cytokines	Oh et al.^29^
NCT03280056	US/Brainstorm	Phase III, double-blind RCT with a sham procedure	Randomized (1:1) (i) Three injections (*n* = 95) (ii) Placebo (*n* = 94) group	Autologous preconditioned BM-MSCs (NurOwn®)	Intrathecal	Safe, did not meet the primary endpoint of efficacy, possible clinical response in subgroups, improvement in biomarkers.	Cudkowicz et al.^38^
NCT04745299	Korea/CorestemChemon	Phase III, double-blind RCT with a sham procedure	Randomized (1:2:2) (i) Two injections with a 26-day interval (ii) Two injections + 3 additional booster injections (iii) Placebo group	Autologous BM-MSCs (Lenzumestrocel, Neuronata-R® Inj)	Intrathecal	Ongoing study	Nam et al.^39^

A minimum set of mandatory items is required to develop an optimal treatment strategy for MSCs in ALS. However, the following limitations of current MSC therapies in ALS should be considered first. (1) Because the recipient’s environment is in a pro-inflammatory state, the injected MSCs would be exposed to the same pathological conditions, which can reduce the capabilities of MSCs. (2) It is difficult for MSCs to replace damaged nerve cells or neural networks. Therefore, repeat treatments with less invasive procedures are necessary to overcome these limitations.

**Figure 2.**
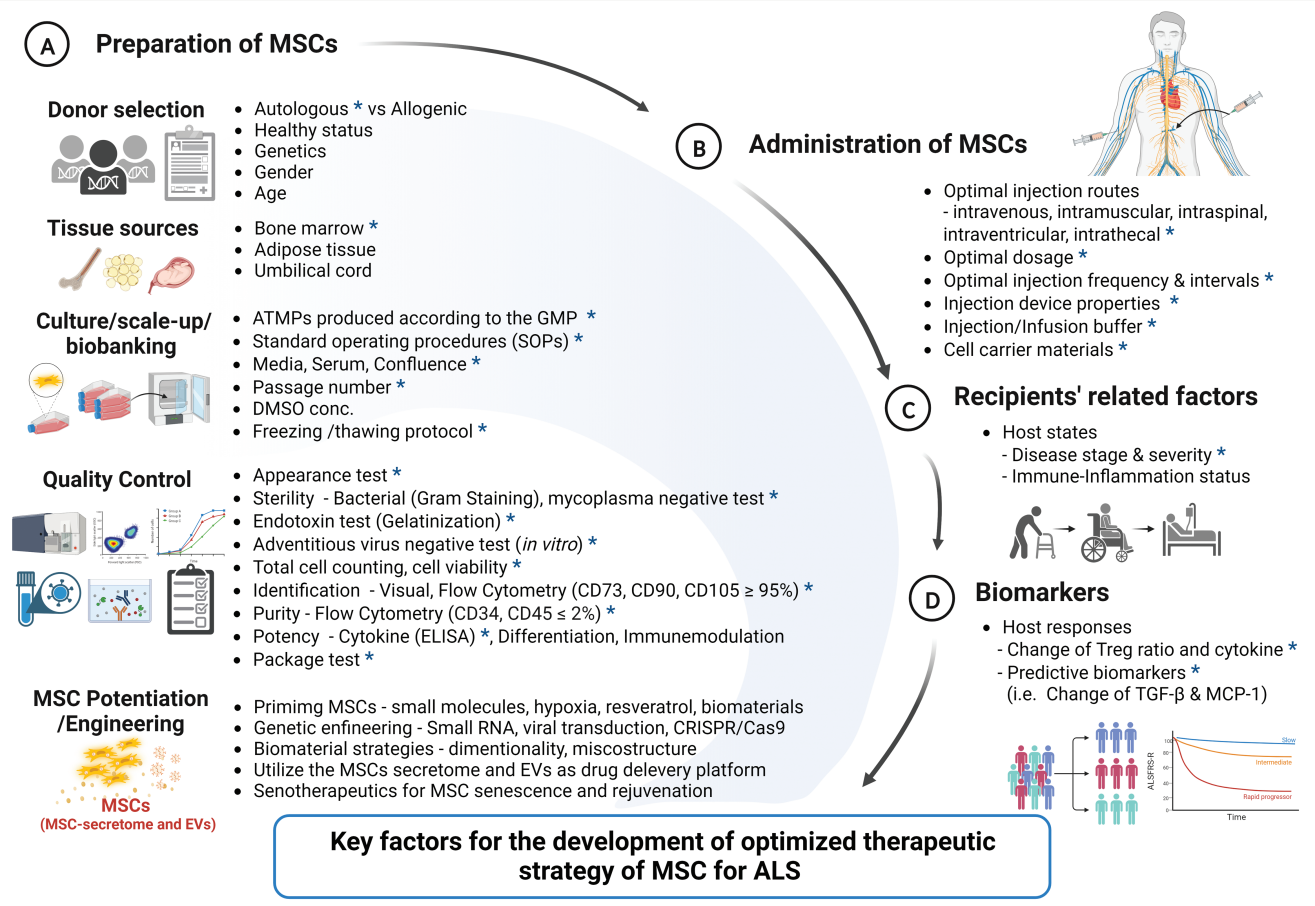
Factors to be considered in the development of an optimal MSC therapeutic strategy for treating ALS. Key factors affecting the clinical outcome of MSC therapy include (**A**) the preparation of MSCs, (**B**) the administration of MSCs, (**C**) recipient-related factors, and (**D**) biomarkers. *Items for preparing and manufacturing the Neuronata-R (lenzumestrocel) product in clinical trials for ALS, which are described in previously published papers.^28,29,39^

The mandatory items can be modified based on a better understanding of the disease and changing perspectives as MSC technology evolves. As shown in Fig. 2, developing safer and more effective BM-MSC therapies for ALS should be considered from 3 perspectives: MSC preparation and manufacturing, administration, and recipient factors.

### Items to be Standardized in MSC Preparation and Manufacturing

Over 40 preclinical and 20 clinical trials using stem cells in ALS have been conducted over 2 decades. However, a detailed GMP manufacturing protocol has yet to be described. Given FDA guidance for ALS drug development^32^ and trial guidelines,^33^ data on quality-controlled MSC products compatible with acceptance criteria for potency and safety issues should be shared between researchers based on the expected mode of action. As shown in Fig. 2, guidelines for MSC preparation and manufacturing as ATMPs produced according to GMP and mandatory quality control (QC) items for each final product should be released to the public as open-source materials.

According to the International Society for Cell and Gene Therapy (ISCT) criteria for MSCs, authentic human MSC-like cells for autologous transplantation must express certain MSC-positive surface markers, including 5ʹ-nucleotidase (CD73), Thy-1 (CD90), and endoglin (CD105) and must lack the expression of macrophage marker CD14, HSC marker CD34, lymphocyte marker CD45, B cell marker CD19, B-cell antigen receptor complex-associated protein alpha chain CD79a, and MHC class II cell surface receptor HLA-DR. In addition to the expression fingerprint, the cells must also be able to the capacity to differentiate into adipogenic, osteogenic, and chondrogenic lineages in vitro.^24^ MSCs secrete bioactive factors that favor tissue remodeling and repair, as well as immunoregulatory properties. These regenerative characteristics of MSCs have collectively made them the most broadly tested adult stem cells in clinical trials. In the case of intrathecal autologous BM-MSCs (Neuronata-R: Lenzumestrocel),^27^ a potency test for QC was conducted following the administration of at least 120 pg of human vascular endothelial growth factor per 1 × 10^4^ cells.^40^ MSC criteria and other items are summarized in Figs. 1 and 2, respectively.

### The Optimal Delivery Route, Dose, and Therapeutic Schedule of MSC Treatment for ALS

One of the critical factors in ALS drug development is the ability of the drug to cross the blood-brain barrier (BBB) and reach the lesion site. The larger the molecule, the more difficult it is to cross the BBB, which is a strong barrier that restricts the passage of cells. In addition to the intrathecal route, MSCs have been administered through a combined intravenous and intrathecal approach for patients with ALS.^41^ While the intravenous route for MSCs has limitations, with a significant portion getting trapped in the lungs, liver, and spleen rapidly after injection,^42^ it remains a more accessible and repeatable administration method. Considering preclinical reports demonstrating the beneficial effects of intravenous MSCs in ALS animal models,^43-45^ the intravenous route continues to be a viable option in ALS. Intrathecal, intracranial, and intraspinal administration are good delivery methods, given the problem of BBB penetration. The intrathecal administration of MSCs is less invasive than intraspinal or intracranial administration and allows for repeat treatments and serial CSF biomarker analysis.

A post hoc survival analysis in the lenzumestrocel phase II trial showed no long-term survival benefit. This finding may have been associated with the small sample size and relatively short observation period in the control group. A pilot surveillance study with a propensity score-matched external control group was conducted to overcome such limitations, including issues related to sample size and observation time.^27^ The survival probability was significantly higher in the BM-MSC group than in the external control group. Additionally, the Cox proportional hazard model showed a statistically significant lower hazard ratio for both single-cycle injection and multiple injections compared to the external control after adjusting for prognostic factors. The survival time of intrathecally delivered MSCs is limited to less than 28 days, but its effectiveness is sustained for up to at least 6 months. Considering the immunomodulatory effects of MSC treatment by intrathecal delivery (less-invasive procedure), whether successive booster MSC treatments after single-cycle treatment could improve long-term efficacy should be determined.

The US FDA and the Korean Ministry of Food and Drug Safety approved the phase III ALSUMMIT clinical trial protocol (NCT04745299). ALSUMMIT is a randomized, multicenter, double-blind, parallel-group, sham procedure-controlled phase III trial to evaluate the long-term efficacy and safety of repeated BM-MSCs in the treatment of ALS (56-week main study with five BM-MSC injections followed by a 24-month observational study). ^39^ All 126 participants were already enrolled, and the ongoing ALSUMMIT trial is expected to have data at the end of 2024. In this protocol, the time interval of booster injections after the single cycle injection was designed to be 3 months. The rationale for 3-month interval booster injections was based on the unpublished data of a post-marketing surveillance study of lenzumestrocel suggesting that CSF cytokine profiles at 3- to 4-month intervals showed more benefits according to ALSFRS-R scores and cytokine levels than those with 5- to 12-month injection intervals. Besides the administration routes, applying a repeated therapeutic protocol at regular intervals is an essential factor for developing an optimal MSC therapeutic strategy for ALS.^27^ Comparative analytic data of serial CSF biomarkers, ALSFRS-R scores, and other clinical parameters could provide vital information to create a more robust MSCs and to design stratified or precision medicine.

The short survival time of injected MSCs could be a limitation of intrathecal MSC therapy but it can be overcome with repeated treatments. However, short life of MSCs can escape from either accumulated toxic effects or unproven fate of MSCs which might be a hazard to the recipient. Therefore, it is crucial to understand the next-generation optimized MSC therapeutic strategy based on lessons from previous MSC trials for ALS.^8,27,34-36^ Thus, the development of optimal clinical protocols, such as less invasive repeated injections with optimized therapeutic intervals based on the characteristics of the intrinsic MSCs intrathecally injected, including the survival time of MSCs in CSF and persisting action duration reflected by biomarker change, is required.

### Recipient Factors

While the precise mechanisms underlying host factors in clinical settings remain unclear, preclinical studies suggest that the disease stage and the surrounding tissue environment can impact the efficacy of MSC therapies. Inflammation, hypoxia, and the extracellular matrix are dynamic in disease progression, and each parameter can influence MSC function in vivo.^46^ In addition, early intervention might be more effective in achieving maximal therapeutic benefits in ALS.^10^

Previous trials^28,29,38^ and a long-term monitoring study^27^ showed that recipient factors, such as older age, rapid progression of the disease, and lower ALSFRS-R scores, were associated with poor autologous BM-MSC treatment outcomes. Younger age and early ALS stage measured by ALSFRS-R scores and an inverse transforming growth factor-β (TGF-β)/monocyte chemoattractant protein-1 (MCP-1/CCL2) ratio after MSC treatment were significant predictors of good prognosis in patients with ALS.^29,38^ Notably, a change in the TGF-β/MCP-1 ratio in CSF, which reflects the patient’s immune-inflammation status, between baseline and after treatment was significantly related to prognosis and long-term outcomes. These findings suggest the importance of host factors and the necessity of a stratification model for the future design of MSC clinical trials. It is challenging to develop a treatment that will be effective for all patients with ALS with varying host factors. Therefore, it is crucial to develop clinical trial designs that consider recipient factors and optimized therapeutic protocols to identify groups of individuals with ALS to maximize treatment benefits.

### Biomarkers

Although MSCs clinical trials for ALS have reported that MCP-1 and TGF-β in CSF might be related to stem cell efficacy,^29^ large-scale clinical trials have not yet been able to objectively evaluate the therapeutic efficacy of MSCs in treating ALS. Plasma phosphorylated neurofilament H was used as a biomarker of efficacy in a clinical trial for the recently approved AMX0035 (Relyvrio), although no significant difference was found after 24 weeks.^5^ In the case of the ALSUMMIT,^39^ a clinical trial is currently underway to identify biomarkers that can evaluate the efficacy of MSCs through CSF analysis at each cycle of administration. The discovery of meaningful biomarkers that reflect the treatment response and efficacy is expected to be an essential factor in new drugs for ALS.

## Conclusion and Future Perspectives

The development of an optimized therapeutic strategy could enhance the efficacy of MSCs and address safety issues. As shown in Fig. 2, various strategies for MSC preparation and manufacturing, including priming or pretreatment methods,^47^ QC protocols, such as selective isolation of healthy stem cells,^48^ genetically modified MSCs with enhanced functionality,^49^ and the application of MSC-derived exosomes,^50^ have been proposed to increase the efficacy of MSC treatment. These strategies may overcome the limitations of the currently proposed autologous BM-MSC therapy for ALS. We suggest that future therapeutic strategies should include the following: (1) protocols for producing optimally primed/engineered MSCs by GMP able to escape from senescence, (2) the development of reliable biomarkers to be used as companion diagnostics for predicting the effectiveness of MSC therapy, and (3) the development of the strategy to overcome host pro-inflammatory milieu that injected MSCs will face.

## Data Availability

No new data were generated or analyzed in support of this research.

## References

[1] Brown RH , Al-Chalabi A. Amyotrophic lateral sclerosis. N Engl J Med. 2017;377(2):162-172. 10.1056/NEJMra1603471 28700839

[2] Hardiman O , Al-Chalabi A, Chio A, et al. Amyotrophic lateral sclerosis. Nat Rev Dis Primers. 2017;20(3):17085. 10.1038/nrdp.2017.85 29052611

[3] Miller RG , Mitchell JD, Lyon M, Moore DH. Riluzole for amyotrophic lateral sclerosis (ALS)/motor neuron disease (MND). Cochrane Database Syst Rev. 2012;2012(3):CD001447. 10.1002/14651858.CD001447.pub3 22419278 PMC7055506

[4] Rothstein JD. Edaravone: a new drug approved for ALS. Cell. 2017;171(4):725. 10.1016/j.cell.2017.10.011 29100067

[5] Paganoni S , Macklin EA, Hendrix S, et al. Trial of sodium phenylbutyrate-taurursodiol for amyotrophic lateral sclerosis. N Engl J Med. 2020;383(10):919-930. 10.1056/NEJMoa1916945 32877582 PMC9134321

[6] Jaiswal MK. Riluzole and edaravone: A tale of two amyotrophic lateral sclerosis drugs. Med Res Rev. 2019;39(2):733-748. 10.1002/med.21528 30101496

[7] Saitoh Y , Takahashi Y. Riluzole for the treatment of amyotrophic lateral sclerosis. Neurodegener Dis Manag. 2020;10(6):343-355. 10.2217/nmt-2020-0033 32847483

[8] Witzel S , Maier A, Steinbach R, et al; German Motor Neuron Disease Network (MND-NET). Safety and effectiveness of long-term intravenous administration of edaravone for treatment of patients with amyotrophic lateral sclerosis. JAMA Neurol. 2022;79(2):121-130. 10.1001/jamaneurol.2021.4893 35006266 PMC8749709

[9] Saez-Atienzar S , Bandres-Ciga S, Langston RG, et al. Genetic analysis of amyotrophic lateral sclerosis identifies contributing pathways and cell types. Sci Adv. 2021;7(3):eabd9036. 10.1126/sciadv.abd9036 33523907 PMC7810371

[10] Mead RJ , Shan N, Reiser HJ, Marshall F, Shaw PJ. Amyotrophic lateral sclerosis: a neurodegenerative disorder poised for successful therapeutic translation. Nat Rev Drug Discov. 2023;22(3):185-212. 10.1038/s41573-022-00612-2 36543887 PMC9768794

[11] Thonhoff JR , Simpson EP, Appel SH. Neuroinflammatory mechanisms in amyotrophic lateral sclerosis pathogenesis. Curr Opin Neurol. 2018;31(5):635-639. 10.1097/WCO.0000000000000599 30048339

[12] Beers DR , Appel SH. Immune dysregulation in amyotrophic lateral sclerosis: mechanisms and emerging therapies. Lancet Neurol. 2019;18(2):211-220. 10.1016/S1474-4422(18)30394-6 30663610

[13] Ferraiuolo L. The non-cell-autonomous component of ALS: new in vitro models and future challenges. Biochem Soc Trans. 2014;42(5):1270-1274. 10.1042/BST20140168 25233402

[14] Beers DR , Henkel JS, Zhao W, et al. Endogenous regulatory T lymphocytes ameliorate amyotrophic lateral sclerosis in mice and correlate with disease progression in patients with amyotrophic lateral sclerosis. Brain. 2011;134(Pt 5):1293-1314. 10.1093/brain/awr074 21596768 PMC3097891

[15] Henkel JS , Beers DR, Wen S, et al. Regulatory T-lymphocytes mediate amyotrophic lateral sclerosis progression and survival. EMBO Mol Med. 2013;5(1):64-79. 10.1002/emmm.201201544 23143995 PMC3569654

[16] Kipnis J , Avidan H, Caspi RR, Schwartz M. Dual effect of CD4+CD25+ regulatory T cells in neurodegeneration: a dialogue with microglia. Proc Natl Acad Sci USA. 2004;101(Suppl 2):14663-14669. 10.1073/pnas.0404842101 15331781 PMC521988

[17] Haimon Z , Frumer GR, Kim JS, et al. Cognate microglia-T cell interactions shape the functional regulatory T cell pool in experimental autoimmune encephalomyelitis pathology. Nat Immunol. 2022;23(12):1749-1762. 10.1038/s41590-022-01360-6 36456736

[18] Hickman S , Izzy S, Sen P, Morsett L, El Khoury J. Microglia in neurodegeneration. Nat Neurosci. 2018;21(10):1359-1369. 10.1038/s41593-018-0242-x 30258234 PMC6817969

[19] Ahani-Nahayati M , Shariati A, Mahmoodi M, et al. Stem cell in neurodegenerative disorders; an emerging strategy. Int J Dev Neurosci. 2021;81(4):291-311. 10.1002/jdn.10101 33650716

[20] Castillo Bautista CM , Sterneckert J. Progress and challenges in directing the differentiation of human iPSCs into spinal motor neurons. Front Cell Dev Biol. 2023;10:1089970. 10.3389/fcell.2022.1089970 36684437 PMC9849822

[21] Jaiswal MK. Therapeutic opportunities and challenges of induced pluripotent stem cells-derived motor neurons for treatment of amyotrophic lateral sclerosis and motor neuron disease. Neural Regen Res. 2017;12(5):723-736. 10.4103/1673-5374.206635 28616022 PMC5461603

[22] Du H , Huo Z, Chen Y, et al. Induced pluripotent stem cells and their applications in amyotrophic lateral sclerosis. Cells. 2023;12(6):971. 10.3390/cells12060971 36980310 PMC10047679

[23] Appel SH , Armon C. Stem cells in amyotrophic lateral sclerosis: ready for prime time? Neurology. 2016;87(4):348-349. 10.1212/WNL.0000000000002906 27358334

[24] Dominici M , Le Blanc K, Mueller I, et al. Minimal criteria for defining multipotent mesenchymal stromal cells The International Society for Cellular Therapy position statement. Cytotherapy. 2006;8(4):315-317. 10.1080/14653240600855905 16923606

[25] Kwon MS , Noh MY, Oh KW, et al. The immunomodulatory effects of human mesenchymal stem cells on peripheral blood mononuclear cells in ALS patients. J Neurochem. 2014;131(2):206-218. 10.1111/jnc.12814 24995608

[26] Noh MY , Lim SM, Oh KW, et al. Mesenchymal stem cells modulate the functional properties of microglia via TGF-beta secretion. Stem Cells Transl Med. 2016;5(11):1538-1549. 10.5966/sctm.2015-0217 27400795 PMC5070497

[27] Nam JY , Chun S, Lee TY, et al. Long-term survival benefits of intrathecal autologous bone marrow-derived mesenchymal stem cells (Neuronata-R(R): lenzumestrocel) treatment in ALS: propensity-score-matched control, surveillance study. Front Aging Neurosci. 2023;15:1148444. 10.3389/fnagi.2023.1148444 37122380 PMC10130504

[28] Oh KW , Moon C, Kim HY, et al. Phase I trial of repeated intrathecal autologous bone marrow-derived mesenchymal stromal cells in amyotrophic lateral sclerosis. Stem Cells Transl Med. 2015;4(6):590-597. 10.5966/sctm.2014-0212 25934946 PMC4449093

[29] Oh KW , Noh MY, Kwon MS, et al. Repeated intrathecal mesenchymal stem cells for amyotrophic lateral sclerosis. Ann Neurol. 2018;84(3):361-373. 10.1002/ana.25302 30048006 PMC6175096

[30] Beers DR , Zhao W, Wang J, et al. ALS patients’ regulatory T lymphocytes are dysfunctional, and correlate with disease progression rate and severity. JCI Insight. 2017;2(5):e89530. 10.1172/jci.insight.89530 28289705 PMC5333967

[31] Kim SH , Oh KW, Jin HK, Bae JS. Immune inflammatory modulation as a potential therapeutic strategy of stem cell therapy for ALS and neurodegenerative diseases. BMB Rep. 2018;51(11):545-546. 10.5483/BMBRep.2018.51.11.255 30463642 PMC6283021

[32] Andrews JA , Bruijn LI, Shefner JM. ALS drug development guidances and trial guidelines: consensus and opportunities for alignment. Neurology. 2019;93(2):66-71. 10.1212/WNL.0000000000007695 31171646 PMC6656654

[33] van den Berg LH , Sorenson E, Gronseth G, et al; Airlie House ALS Clinical Trials Guidelines Group. Revised Airlie House consensus guidelines for design and implementation of ALS clinical trials. Neurology. 2019;92(14):e1610-e1623. 10.1212/WNL.0000000000007242 30850440 PMC6448453

[34] Morata-Tarifa C , Azkona G, Glass J, Mazzini L, Sanchez-Pernaute R. Looking backward to move forward: a meta-analysis of stem cell therapy in amyotrophic lateral sclerosis. NPJ Regen Med. 2021;6(1):20. 10.1038/s41536-021-00131-5 33795700 PMC8016966

[35] Sironi F , De Marchi F, Mazzini L, Bendotti C. Cell therapy in ALS: an update on preclinical and clinical studies. Brain Res Bull. 2023;194:64-81. 10.1016/j.brainresbull.2023.01.008 36690163

[36] Abdul Wahid SF , Law ZK, Ismail NA, Lai NM. Cell-based therapies for amyotrophic lateral sclerosis/motor neuron disease. Cochrane Database Syst Rev. 2019;12(12):CD011742. 10.1002/14651858.CD011742.pub3 31853962 PMC6920743

[37] Petrou P , Gothelf Y, Argov Z, et al. Safety and clinical effects of mesenchymal stem cells secreting neurotrophic factor transplantation in patients with amyotrophic lateral sclerosis: results of phase 1/2 and 2a clinical trials. JAMA Neurol. 2016;73(3):337-344. 10.1001/jamaneurol.2015.4321 26751635

[38] Cudkowicz ME , Lindborg SR, Goyal NA, et al. A randomized placebo-controlled phase 3 study of mesenchymal stem cells induced to secrete high levels of neurotrophic factors in amyotrophic lateral sclerosis. Muscle Nerve. 2022;65(3):291-302. 10.1002/mus.27472 34890069 PMC9305113

[39] Nam JY , Lee TY, Kim K, et al. Efficacy and safety of Lenzumestrocel (Neuronata-R(R) inj) in patients with amyotrophic lateral sclerosis (ALSUMMIT study): study protocol for a multicentre, randomized, double-blind, parallel-group, sham procedure-controlled, phase III trial. Trials. 2022;23(1):415. 10.1186/s13063-022-06327-4 35585556 PMC9115933

[40] Kim HY , Kim H, Oh KW, et al. Biological markers of mesenchymal stromal cells as predictors of response to autologous stem cell transplantation in patients with amyotrophic lateral sclerosis: an investigator-initiated trial and in vivo study. Stem Cells. 2014;32(10):2724-2731. 10.1002/stem.1770 24966156

[41] Tavakol-Afshari J , Boroumand AR, Farkhad NK, et al. Safety and efficacy of bone marrow derived-mesenchymal stem cells transplantation in patients with amyotrophic lateral sclerosis. Regen Ther. 2021;18:268-274. 10.1016/j.reth.2021.07.006 34466632 PMC8377537

[42] Fischer UM , Harting MT, Jimenez F, et al. Pulmonary passage is a major obstacle for intravenous stem cell delivery: the pulmonary first-pass effect. Stem Cells Dev. 2009;18(5):683-692. 10.1089/scd.2008.0253 19099374 PMC3190292

[43] Uccelli A , Milanese M, Principato MC, et al. Intravenous mesenchymal stem cells improve survival and motor function in experimental amyotrophic lateral sclerosis. Mol Med. 2012;18(1):794-804. 10.2119/molmed.2011.00498 22481270 PMC3409288

[44] Magota H , Sasaki M, Kataoka-Sasaki Y, et al. Intravenous infusion of mesenchymal stem cells delays disease progression in the SOD1G93A transgenic amyotrophic lateral sclerosis rat model. Brain Res. 2021;1757:147296. 10.1016/j.brainres.2021.147296 33516815

[45] Magota H , Sasaki M, Kataoka-Sasaki Y, et al. Repeated infusion of mesenchymal stem cells maintain the condition to inhibit deteriorated motor function, leading to an extended lifespan in the SOD1G93A rat model of amyotrophic lateral sclerosis. Mol Brain. 2021;14(1):76. 10.1186/s13041-021-00787-6 33962678 PMC8103621

[46] Shi Y , Wang Y, Li Q, et al. Immunoregulatory mechanisms of mesenchymal stem and stromal cells in inflammatory diseases. Nat Rev Nephrol. 2018;14(8):493-507. 10.1038/s41581-018-0023-5 29895977

[47] Li M , Jiang Y, Hou Q, et al. Potential pre-activation strategies for improving therapeutic efficacy of mesenchymal stem cells: current status and future prospects. Stem Cell Res Ther. 2022;13(1):146. 10.1186/s13287-022-02822-2 35379361 PMC8981790

[48] Zhou X , Hong Y, Zhang H, Li X. Mesenchymal stem cell senescence and rejuvenation: current status and challenges. Front Cell Dev Biol. 2020;8:364. 10.3389/fcell.2020.00364 32582691 PMC7283395

[49] Ocansey DKW , Pei B, Yan Y, et al. Improved therapeutics of modified mesenchymal stem cells: an update. J Transl Med. 2020;18(1):42. 10.1186/s12967-020-02234-x 32000804 PMC6993499

[50] Nikfarjam S , Rezaie J, Zolbanin NM, Jafari R. Mesenchymal stem cell derived-exosomes: a modern approach in translational medicine. J Transl Med. 2020;18(1):449. 10.1186/s12967-020-02622-3 33246476 PMC7691969

